# Current Developments in Diagnosis of Salivary Gland Tumors: From Structure to Artificial Intelligence

**DOI:** 10.3390/life14060727

**Published:** 2024-06-05

**Authors:** Alexandra Corina Faur, Roxana Buzaș, Adrian Emil Lăzărescu, Laura Andreea Ghenciu

**Affiliations:** 1 Department of Anatomy and Embriology, ”Victor Babeș” University of Medicine and Pharmacy, Eftimie Murgu Square, No. 2, 300041 Timișoara, Romania; faur.alexandra@umft.ro (A.C.F.); lazarescu.adrian@umft.ro (A.E.L.); 2 Department of Internal Medicine I, Center for Advanced Research in Cardiovascular Pathology and Hemostaseology, ”Victor Babeș” University of Medicine and Pharmacy, Eftimie Murgu Square, No. 2, 300041 Timișoara, Romania; 3 Department of Functional Sciences, ”Victor Babeș”University of Medicine and Pharmacy, Eftimie Murgu Square, No. 2, 300041 Timișoara, Romania; bolintineanu.laura@umft.ro

**Keywords:** salivary gland tumors, classification, immunohistochemistry, artificial intelligence

## Abstract

Salivary glands tumors are uncommon neoplasms with variable incidence, heterogenous histologies and unpredictable biological behaviour. Most tumors are located in the parotid gland. Benign salivary tumors represent 54–79% of cases and pleomorphic adenoma is frequently diagnosed in this group. Salivary glands malignant tumors that are more commonly diagnosed are adenoid cystic carcinomas and mucoepidermoid carcinomas. Because of their diversity and overlapping features, these tumors require complex methods of evaluation. Diagnostic procedures include imaging techniques combined with clinical examination, fine needle aspiration and histopathological investigation of the excised specimens. This narrative review describes the advances in the diagnosis methods of these unusual tumors—from histomorphology to artificial intelligence algorithms.

## 1. Introduction

The parotid, submandibular, sublingual and minor salivary glands have the function of producing saliva. Saliva is important for the digestive system because it facilitates mastication, swallowing, digestion, teeth remineralization and has antimicrobial properties [1]. Sometimes, neoplastic lesions can affect the salivary glands. Therefore, it is critical for the physician to have knowledge of the diagnostic processes involved in the evaluation of head and neck lesions, especially those of the salivary glands. Salivary gland tumors constitute a heterogeneous group of neoplasms with a variable incidence and a challenging diagnosis. Recent data show that salivary tumors represent 3–10% of neoplasms in the head and neck region and 0.3% of human neoplasms [2,3,4,5,6]. However, their incidence ranges geographically (between 0.4 and 13.5% reported incidence) [5,6]. Primary salivary gland tumours are located in most cases in the parotid gland (64–80%) and in the submandibular gland (7–15%). Twenty-five percent of parotid tumors are malignant. Between 1 and 23% of cases are found in the sublingual gland and minor salivary glands and are mostly malignant [1,5,6,7,8,9,10,11,12,13,14].

Benign salivary tumors represent 54–79% of cases, while 12–46% are malignant. The more frequently diagnosed benign salivary tumors are pleomorphic adenomas (65%). In the group of malignant salivary gland tumors, adenoid cystic carcinomas (ACCs) and mucoepidermoid carcinomas (MECs) are more commonly reported [1,5,6,7,8,9,10,11,12]. Surgery is the treatment of choice for salivary gland tumors. Additional postoperative radiotherapy is needed in some cases. Furthermore, patients with advanced disease can receive preoperative chemotherapy to reduce the tumor size. Because of the variable biological behaviour of salivary tumors and the reduced number of cases that can be analyzed in one center, the evolution of these cases is unpredictable. Salivary gland neoplasms show a wide range of histological diversity and overlapping features that require complex methods of evaluation. The diagnostic procedures include imaging techniques combined with clinical examination and fine needle aspiration cytology. Molecular targets are now tested for improving treatment and diagnosis. Computer-aided diagnosis tools are in development to assist doctors in the interpretation of medical images. However, additional testing may have economic implications, requires specific training and is more frequently available in specialized centers [4,12]. In this paper, we have made a narrative review of the advances in the diagnosis methods of these rare tumors.

## 2. Morphological Diagnosis of Salivary Neoplasms

### 2.1. New Concepts in Classification and Grading of Salivary Gland Tumors

**Figure 1:**
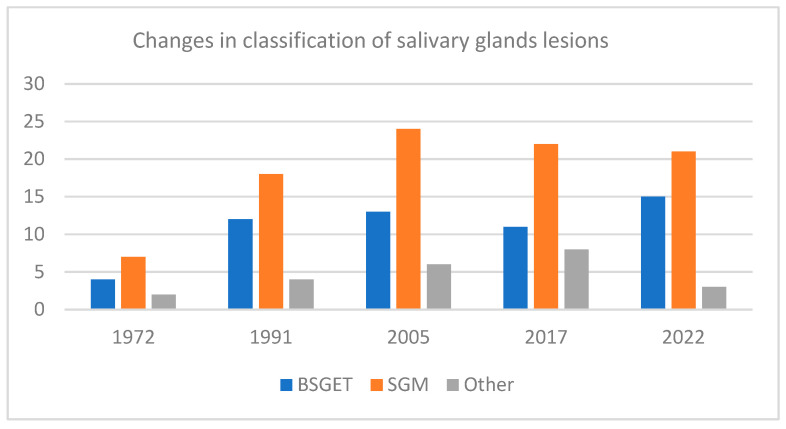
Salivary gland entities reported in WHO from 1972 to 2022 [19]. BSGE = salivary gland benign epithelial tumors; SGM = salivary gland malignancies; Other = non-epithelial, soft tissue, lymphoid, secondary or unclassified salivary gland entities.

The World Health Organization (WHO) classification of head and neck tumors of salivary glands is an ever-evolving process because of the complex histopathological appearance that these tumors have [15,16,17,18,19]. The 2005 classification system for salivary tumors listed 24 malignant tumors and 10 benign ones as major categories. The benign tumors represented by ductal papilloma (DP) and cystadenoma (CyA) have subfield divisions. The 2017 classification removed some types and introduced new entities. Here, the poorly differentiated carcinoma is also divided into undifferentiated carcinoma (UC), large cell neuroendocrine carcinoma and small cell neuroendocrine carcinoma. New malignant salivary glands entities were introduced in the latest fifth edition of the WHO classification (2022): microsecretory carcinoma (MS) and sclerosing microcystic adenocarcinoma (SMcADK). In the benign salivary neoplasm category keratocystoma (KC), intercalated and striated duct adenomas (InDA and SDA) were introduced [16,17]. Overall, 11 to 36 entities were reported from 1972 to 2022 but there are still controversies as to how the classification of primary salivary glands tumors should be conducted [15,16,17,18,19]. The evolution of the salivary gland entities reported in WHO over the years is summarized in Figure 1.

The evolution of these classifications and the introduction of new terms are important aspects for a better understanding of salivary gland lesion diagnosis. Now the benign salivary tumors have lymphadenomas (LA) and DP categories. Tumors previously known as sebaceous and non-sebaceous lymphadenomas are now classified only as LAs. LAs are the biphasic benign tumors with lymphoid stroma and epithelial components represented by myoepithelial, squamous and ductal cells with/without sebaceous elements. The malignant transformation of these tumors is rare and considered as sebaceous lymphadenocarcinoma (LyC). The DP are intraductal epithelial proliferations with transitional or bland columnar type cells that in past classification systems were known as inverted ductal and intraductal papillomas. Canalicular adenoma (CA) remains as a singular term. Metastasing pleomorphic adenoma is considered to be a pleomorphic adenoma (PA) with aggressive behaviour that has to be treated with caution but is still a part of the benign group of salivary tumors [8,17]. Sialoblastoma (SB) is classified as a tumor with uncertain malignant potential [18].

The malignant salivary gland tumor classification has broadened the adenocarcinoma not otherwise specified (ADK NOS) category with the inclusion of cystadenocarcinoma, papillary cystadenocarcinoma, sclerosing microcystic adenocarcinoma, microsecretory carcinoma and mucinous adenocarcinoma. [8]. Some authors’ reports showed that poorly differentiated carcinomas, adenocarcinomas NOS and carcinomas with oncocytic cells can be included in salivary carcinomas NOS and classified as emerging entities [19]. Mucinous adenocarcinoma is now recognized as being related to salivary intraductal papillary mucinous neoplasms and is subdivided into papillary, colloid and signet-ring adenocarcinomas [16]. Polymorphous low-grade adenocarcinoma (PLGADK) was shortened to polymorphous adenocarcinoma (PADK). This category is the most debated one. PLGADK was the term used to describe a low grade, cribriform carcinoma of minor salivary glands. Because 90% of PADKs are diagnosed in minor salivary glands (especially those of the tongue), some authors have used the term cribriform adenocarcinoma of the minor salivary glands for these entities. Histologically, the polymorphous features include a lobular pattern of a widely infiltrative tumor with single cells with cells arranged in a targetoid appearance around the nerves, but also with tubular, fascicular and papillary architecture [18]. Over the years, aggressive tumors of this histological type were reported, so the removal of the “low-grade” from its name was proposed and accepted from 2017. By this removal, the polymorphous adenocarcinoma treatment is the same as for a malignant salivary gland tumor [7,8]. The PADK has to be differentiated from the ACC and PA, and here ancillay immunohistochemical tests for p40 and ΔNp63 can be helpful [17]. 

There is a new field of salivary carcinoma NOS and emerging entities in which oncocytic carcinoma (OncC) and poorly differentiated carcinoma are included [19]. ACC, carcinoma ex pleomorphic adenoma (CEPA), MEC and acinic cell carcinoma (AclC) are terms that have remained the same over the years. However, there are some aspects that have evolved. MEC and AclC were initially considered tumors with borderline behaviour and classified as malignant tumors in the 1991 WHO classification. The 1972 term for carcinoma in pleomorphic adenoma was updated in 2005 as carcinoma ex pleomorphic adenoma. Furthermore, in the last editions, it is specified that the histological type of the CEPAs carcinomatous component has to be reported [15,16,17,18,19].

The grading of MEC remained for low, intermediate and high-grade tumors, but the grading criteria and specific scheme are still lacking [17,20]. Grading systems for ACCs, based upon their morphological appearances, were proposed [19,20,21]. However, these grading schemes are considered useful for assessing prognosis and not in therapeutic management. CEPA are to be graded according to the malignant component [21]. 

Identifying poorly differentiated or high-grade components in otherwise low-grade carcinoma is of paramount interest for the evolution of the patients. The “high-grade transformation” term is preferred over the “dedifferentiation” that was used earlier. The histological types that more frequently show aspects of high-grade transformation are considered to be AclA, ACC, MEC, myoepithelial carcinoma (MC), and epithelial-myoepithelial carcinoma (EMC) [17,22,23,24,25]. Rarely, secretory carcinoma (SC) and PADK cases also develop aspects of high-grade transformation [17]. Recognition of high-grade transformation of salivary gland tumors is important since the patient has a poorer prognosis and needs a more aggressive clinical treatment [15,22,26].

Intraductal carcinoma (IC) of the salivary glands is morphologically similar to low-grade ductal carcinoma in situ or atypical hyperplasia of the breast. Salivary gland entities of these carcinomas were known as low-grade cribriform cystadenocarcinoma, low-grade salivary duct carcinoma and salivary duct carcinoma (SDC). Cystic tumors with cribriform architecture (micropapillary or solid) are regarded today as intraductal carcinoma if invasion is absent [27]. The current grading of the IC is into low and high-grade categories. ICs with monomorphic cells, bland-looking nuclei, scant cytoplasm and delimited by immunopositive cells for myoepithelial markers are of low grade. If features like necrosis, atypia and mitoses are encountered, then it is a high-grade IC. The sampling of the high-grade intraductal carcinoma is important. Such a lesion has to be thoroughly sampled to exclude invasion. [8,27,28].

### 2.2. Advances in the Immunohistochemical Analysis in Salivary Gland Tumors with Morphological Correlations

In the literature there are studies whose results have shown that the immunohistochemical profiles of cytokeratines (CK) 7 and 20 may be useful in the differential diagnosis between primary salivary gland tumors and squamous cell carcinomas and/or tumor metastases in the salivary glands [8,29,30,31]. For the salivary glands, neoplasms the CK7+/CK20− imunoprofile are considered to be characteristic. In the study by Meer S and Altini M, all salivary gland tumors included in the study presented this profile [32]. Another study reported that all the SDCs were negative for CK 20 (both intraductal types and invasive) [27]. The nine types of salivary carcinomas studied by Nikitakis GN et al. were all CK7 immunoreactive [33]. The CK7 and CK20 were useful in identifying the origin of two cases of adenocarcinoma on cell blocks as being tumors of the parotid gland [34].

Immunopositivity for GATA3 in a head and neck tumor is suggestive of a salivary gland origin of the tumor process. In SCD and SC, large areas of tumor cell nuclei that were intensely positive for GATA 3 were noted in all cases subjected to immunohistochemical examination. On restricted areas of weak immunopositivity, immunolabeling for GATA3 was observed in tumors such as AclCs, EMCs, ACCs, MECs and OncCs. Benign salivary tumors such as oncocytomas (Onc), PA and Warthin tumors (WT) exhibit this pattern of immunolabelling as well. Special attention regarding immunopositivity for GATA3 should be given in the differential diagnosis between SDC and high-grade salivary MEC, as both tumor entities can be positive in large areas for this marker [35]. However, there are not many studies researching the GATA 3 imunoprofile of the salivary gland tumors. Careful study of the histology of the tumor can establish the diagnosis; thus, in SDC a cribriform architecture and comedonecrosis are identified, while in high-grade MEC the tumor cells have squamous characteristics and contain secretory material [35].

Another study analyzed the expression of p63, p73 and p53 in benign salivary gland tumors with similar morphology, and basal and myoepithelial cells as components. In PAs p63 and p73, expression was positive in the splindle and myoepithelial cells, with only two cases p53 immunopositive. All the 12 cases of myoepitheliomas studied were positive for p63, 10 cases showed immunopositivity for p73, and only 3 cases had p53 positive cells. Basal cell adenomas (BA) were p63 and p73-positive and negative for p53. Oncs and CAs were negative for p53 and only focal cells were positive for p63 and p73 [36].

SCD and ACC are two representative examples of salivary tumors with a cribriform structure. Cribriform areas have also been reported in BA, PA, EMC, PADK, low-grade cribriform cystadenocarcinoma (LGCADK), basal cell adenocarcinoma (BADK) and SB [8,9,16,17,18,19,20,29]. The differentiation algorithm of Nagao T et al. initially included a myoepithelial marker and then immunolabeling for *Ki-67* and *S-100* protein [29]. A cut-off value for *Ki-67* of 10% was used. Tumors positive for smooth muscle actine/calponin with a *Ki-67* ≤ 10% labelling index can have a diagnosis of PA, BA, PADK and EMC. The PA is further positive for glial fibrillary acidic protein (GFAP). In the category of tumors showing positivity for smooth muscle actine/calponin and *Ki-67* ≥ cu 10%, the following types should be included: ACC, BA, EMC and SB. In this study, the tumors immunopositive for *S-100* were diagnosed as low-grade pleomorphic adenocarcinoma or low-grade cribriform cystadenocarcinoma. In these authors’ immunohistochemistry-based algorithm, if the *S-100* marker is negative, the presumptive diagnosis should be of a SDC [29]. In cases with malignant tumors, it is necessary to identify the perivascular and perineural infiltrative aspect, the presence of necrosis and a high mitotic rate. However, an increased rate of proliferation demonstrated using the *Ki-67* index (>5%) correlates with an increased immunopositivity for p53, and EGFR and the loss of expression for Bcl-2 are aspects that rather support a diagnosis of carcinoma than a benign tumor [29,30].

The results of some studies have shown the usefulness of the immunohistochemical marker GFAP in the differential diagnosis between PA and PADK, the adenocarcinoma cells being immunonegative for this marker [37,38]. Using this information, Curran et al. investigated GFAP expression on tumor fragments of PA, CA and PLGADK of minor salivary glands. All of the PAs studied and 96% of the CAs were positive for GFAP with intensely immunopositive tumor cells located at the interface of the tumor with the connective tissue. All the PADK showed weak or absent intralesional immunopositivity and no peripheral immunoreactivity [37]. GFAP is considered a potential marker for diagnosing tumors with cartilaginous differentiation [38]. However, GFAP and DOG-1 immunoreactivity was described in PAs and BAs [39].

**Figure 2:**
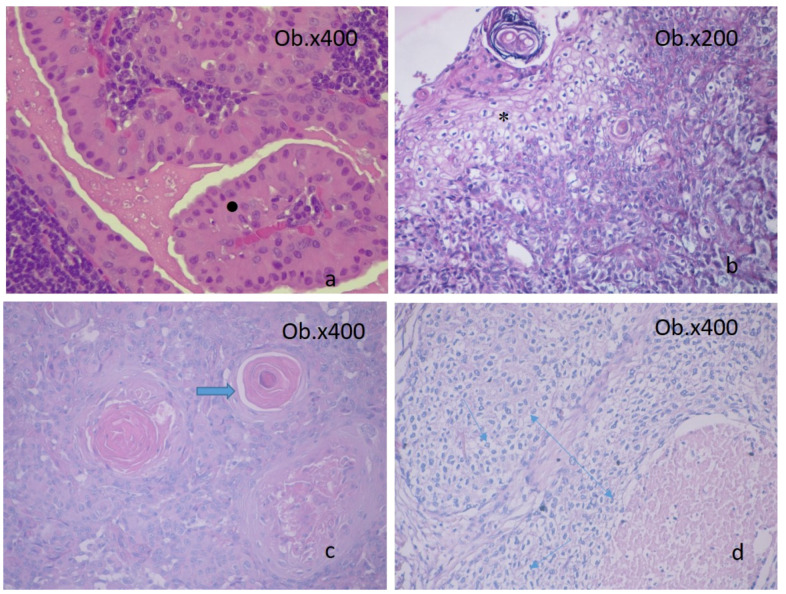
Examples of cells in salivary gland tumors: (**a**) Warthin tumor with ococytes ●; (**b**) PA with sebaceus-looking cells *; (**c**) PA with squamous cells (big arrow); (**d**) SCD with clear cells (arrows); Ob = objective (images obtained using Leica DM750 microscope with digital camera, 200× and 400× magnification).

In salivary gland tumors, proliferating cells may have ductal or myoepithelial differentiation and may present areas with oncocytic, sebaceous, squamous or clear cells (Figure 2). 

The ductal cells are immunohistochemically positive for epithelial membrane antigen (EMA) and carcinoembryonic antigen (CEA). Squamous cells, basal cells and myoepithelial cells of salivary neoplasms test positive for basal type cytokeratin 14 (CK14) and p63. Basal cells and myoepithelial cells are negative for EMA and CEA. For the myoepithelial differentiation, useful markers are *S-100*, α-smooth muscle actin (SMA), muscle-specific actin (MSA), podoplanin, vimentin and calponin. Oncocytic cells test positive for anti-mitochondria antibodies. Sebaceous cells show positive stains for EMA, adipophilin and perilipin. The staining pattern of the myoepithelial markers is also different. In the spindle cells, the staining pattern for SMA, MSA and calponin is diffuse; between the epithelioid cells and those with clear cytoplasm, positive cells can be identified but only in limited areas; the plasmacytoid cells are mostly calponin-positive but are negative for SMA and MSA [29]. Summarizing the literature data [23,24,25,26,27,28,29,30,31,32,33,34,35,36,37,38,39,40,41,42,43,44,45], correlations can be noted between the morphology of the tumors and the immunohistochemical examination. Those correlations are presented in Table 1.

In CEPA, the immunohistochemical analysis is complex and differs depending on the subtype of carcinoma that develops in the existing PA. Some markers are considered specific for the analysis of some tumors. Most PAs are nuclear positive in cells in areas with cartilaginous and myoepithelial differentiation for PLAG1. The expression for Myb is considered useful for the identification of ACC (being identified in 68–82% of cases). GFAP-15, human epidermal growth factor (HER2) and androgen receptors (AR) are frequently positive markers in salivary duct carcinomas, but some authors consider their specificity questionable [7,8,16]. The marker considered useful for appreciating the difference between the conventional and the high-grade transformation component of a salivary gland tumor is *Ki-67*, since in all studied salivary tumors an increased proliferation index was detected in the transformed component compared to the conventional one [29,42]. In Table 2 and Table 3 we have presented the current literature data of the immunohistochemical markers considered useful in salivary gland tumor diagnosis [3,4,5,6,7,8,9,23,24,25,26,27,28,29,30,31,32,33,34,35,36,37,38,39,40,41,42,43,44,45,46,47,48,49].

### 2.3. Genetic Alterations

Genetic alterations are associated with salivary gland neoplasias. Tumor-specific chromosomal rearrangement implicated in the tumorigenesis of specific types of salivary glands tumors are considered to be pathognomonic and useful for diagnosis. Chromosomal translocation at 8q12 resulting in PLAG-1 gene fusion/amplifications are the commonly described genetic aberrations in patients with PA and CEPA. Cases with chromosomal rearrangement in the chromosome 12q14-15 with HMGA-2 fusion are also described for these two types of salivary tumors. In ACC, genetic translocation of the MYB gene to the transcription factor gene NIFIB has as a result the MYB-NFIB fusion, an oncogene considered to be an important factor for tumor proliferation [3,50]. MECs are frequently associated with chromosomal rearrangement t (11; 19) (q21; p13), and alterations of CRTC1-MAML2 are described in 40–90% of these cases [16]. Microsecretory adenocarcinoma was identified as a newly discovered salivary gland tumor via molecular studies that showed a low-grade adenocarcinoma with a specific MEF2C:SS18 fusion [51]. For the secretory carcinoma, the fusion of ETV6 with NTVK3 is considered typical for this type of salivary tumor. The ETV6-NTVK3 fusion gene promotes cell proliferation and is a result of the translocation t (12; 15) (p13; q15). Mucinous adenocarcinoma is characterized by AKT1 E17 K mutations [16]. Rearrangement of the PRKD 1-3 described in PADK and the HTN3-MSANTD3 fusion reported in AclC are found exclusively in these types of tumors [20]. The distinctive histopathological aspect and the EWSR1-ATF1 fusion are considered diagnosis-defining features for hyalinising CCC [52]. Patients with Brooke-Spieger syndrome present abnormalities at the level of chromosome 16q12-q13 and are characterized by the development of skin and salivary glands tumors. The patients with this syndrome develop numerous cutaneous cylindromas, trichoepitheliomas, eccrine spiradenomas with an appearance similar to membranous basal cell adenoma (dermal analogue), and (rarely) salivary glands neoplasms [53]. Other salivary tumors recognized by some studies as having familial aggregation are PA, AclC, WT and LyC. In addition, LyC is associated with trichoepitheliomas in Finns [3,50]. However, none of these molecular alterations are regarded as necessary for the diagnosis [17]. Furthermore, some genetic alterations are not specific for just one histopathological type. For example, PLAG1 fusion is described in AP, CEPA and MC. HRAS mutation is reported in EMC, SCD and IC apocrine subtype [19].

### 2.4. Fine-Needle Aspiration

Fine-needle aspiration (FNA) is included in the pre-surgery workup for salivary gland neoplasm diagnosis. In 2018, the Milan System for Reporting Salivary Gland Cytopathology (MSRSGC) was published. This system provided six diagnosis categories for salivary gland lesions: non-diagnostic, non-neoplastic, atypia of undetermined significance (AUS), neoplasm, suspicion for malignancy and malignant. The purpose of MSRSGC is to offer an easy-to-use guide for cytopathologists. Furthermore, each of the diagnostic categories is associated with a risk of malignancy (ROM) based on literature reviews and treatment recommendations. A second edition of MSRSGC was provided in 2023. In this new edition, there are some changes in the diagnostic category chapters. For the non-diagnostic category, a minimal number of lesional cells for a satisfactory aspirate is no longer required. The literature-based ROM for this category was changed from 25% to 15%. The ROM for the non-neoplastic category is 11% (from 10% in the first edition) and more salivary gland lesions are discussed. The AUS category was kept. In the literature studies, AUS was reported by 0 to 73% of the cases (highly variable). The level of ROM in the benign neoplasm category is <3 but in the suspicion of malignancy category it was raised to 83% (from 60%). In the malignant category, the refined ROM is now >98 and the differential diagnosis and the cytologic features of the most common salivary gland neoplasms likely to be sampled by FNA are provided. This system has improved the communication of data between doctors of different specialties. However, FNA cytology accuracy is considered to be more reliable in the diagnostic of benign salivary gland tumors which pathologists encounter frequently, like pleomorphic adenomas and Warthin tumors [5,54,55,56].

### 2.5. Imaging Diagnosis

Ultrasound, computed tomography (CT) and magnetic resonance imaging (MRI) are available to study these lesions. Ultrasound is affordable and can evaluate tumor borders and content, but it poorly visualizes the deep lobe lesions of the parotid gland. The CT technique involves radiation, and salivary gland tumors can be similar in its images. Magnetic resonance imaging has high resolution but also involves high costs [57,58,59].

The combination between non-contrast MRI with diffusion-weighted imaging, arterial spin labelling and amide proton transfer weighted imaging was shown to be able to differentiate between benign and malignant salivary gland lesions [60]. Positron emission tomography (PET) with a 4′-[methyl-11C]-thiothymidine tracer showed intense uptake in parotid carcinoma and Warthin’s tumors [61]. Recent studies have used ^68^Ga-radiolabeled fibroblast activation protein inhibitor (FAPI) PET/CT to detect head and neck cancer of unknown primary with intensive uptake in the submandibular gland [62]. FAPI-PET imaging was used to investigate adenoid cystic carcinoma target area contours and prognosis, with promising results [63].

The method of choice for investigating the superficial lesion of the parotid gland in children and pregnant women is sonography. CT is used mostly in patients with suspected inflammatory disease, while MRI is the method of choice for in patients with neoplastic lesions [64].

## 3. Artificial Intelligence Algorithms in Salivary Gland Tumor Diagnosis

Several diagnostic methods are currently studied for establishing diagnosis and guiding the treatment strategy for salivary gland tumors. Morphological parameters like inflammatory biomarkers and radiomics extracted from imaging techniques or from histopathological slides are analyzed as possible targets in establishing diagnosis [61,65].

Today, a significant number of medical studies focus their research methods on artificial intelligence (AI). AI is a broad term used to define computer algorithms similar to human brains. AI is expected to reduce human error, save time, improve objectivity, identify hidden data and enhance the workflow in the laboratory. Machine learning (ML) is considered a subfield of AI. ML represents the ability of a computer to learn by applying mathematical algorithms to recognize patterns. The inputs for these ML can be data represented by age, weight, gender, features considered risk factors, gross aspects, anatomical landmarks or histopathological slides [66,67].

AI-assisted salivary biomarker models for oral cancer diagnosis are now being studied [68]. However, the research field of AI-driven salivary gland tumor analysis is still in development mostly due to the limited number of cases in the same center of diagnosis [69]. A reduced number of parotid gland tumors (ranging from 25 to 293) analyzed with AI algorithms have been reported in the recent literature [57,61,68,69,70,71,72,73,74]. All the data of these studies are examined retrospectively. Deep learning algorithms combined with anomaly detection method were built to differentiate between the magnetic resonance images (MRI) of benign and malignant salivary parotid tumors [70]. Studies have shown that deep learning algorithms outperformed physicians in some cases [71]. Matrix-assisted laser desorption/ionization imaging (MALDI) was used together with artificial neural networks to perform an automated deep learning classification of adenoid cystic carcinoma as a subgroup of salivary gland tumors [72]. Some authors have proposed a two-dimensional convolution neural network U-Net model for identifying Warthin tumors and pleomorphic adenomas of the parotid gland on MRI [73].

Currently, it is considered that AI could be an efficient assistant to the pathologist in a variety of digital pathologist tasks. For analyzing histopathological slides, the important feature to develop in deep learning is to enable computers to automatically extract features from the images and build an algorithm of diagnosis. For the pathological diagnosis, the digitized imaging of slides (represented by static images of individual fields-of-view) and whole-slide imaging are used by the new branch of pathology—computational pathology [75]. Deep learning methods, mostly of convolutional neural networks, have been used as computational pathology techniques for the analysis of images of bladder, lung, brain, breast, skin, digestive and genital tumors. These techniques can also integrate into their assessment demographic and molecular data and prognostic and treatment outcomes [71]. Digital pathology systems such as Philips and Leica Biosystems Aperio AT2 DX have been used in clinical trials to compare the diagnostic performance of digital pathology and conventional microscopy [68].

**Figure 3:**
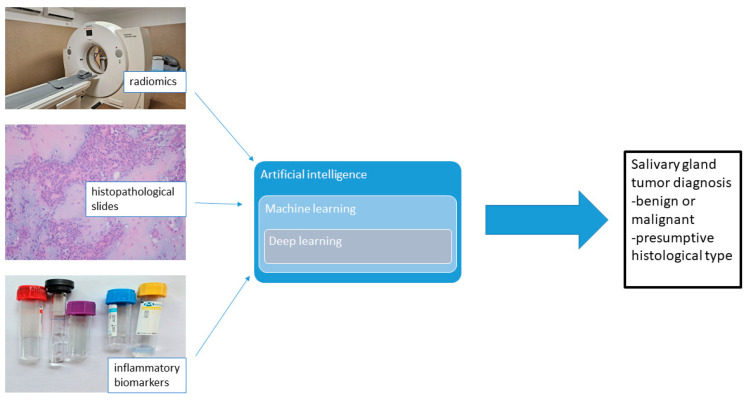
AI algorithms for the classification of salivary glands tumor type.

The use of digital pathology images has its advantages. These include remote consultations, triage of cases to prioritize neoplasms or identify improper tissue samples, higher efficiency with reduced diagnosis time and interpathologist variability [67,74,76,77,78]. Figure 3 summarizes the workflow presented in the previously described studies regarding the AI algorithms in salivary gland tumor assessment.

## 4. Reporting Salivary Gland Malignant Tumors

In the dataset report of a salivary gland carcinoma, the following aspects must be present: the type of surgical procedure, the site (salivary gland), tumor focality, tumor dimension, tumor type according to the latest classification system, tumor grading (for those who have a system to fit in), presence and extent of invasion, status of the surgical margin and pathologic staging. It is necessary to specify the topography of the lesion. The unspecified topography category is considered an exception. If present, the high-grade transformation has to be specified in the pathology report. Elements such as coexisting pathology and ancillary tests can also be disclosed for a better understanding of the salivary neoplasm’s biology [18].

For the establishment of a pathology report for a patient with salivary gland cancer, the collaboration between surgeon and pathologist is considered crucial [18]. The tumor size should be measured before formalin fixation. It is known that with formalin fixation, the specimen shrinks significantly [79]. Another useful element is considered to be the assessment of the status of the excision margins. Here we can see some differences. Thus, an appropriate and useful excision margin in salivary tumor surgery falls within the average of 5–6 mm. However, there are data that have shown that 20% of adenoid cystic salivary gland carcinomas recur even if they were excised with a margin of excision greater than 5 mm. Others, however, claim that salivary tumors classified as having a similar evolution to low-grade tumors, such as epithelial-myoepithelial carcinoma, are cured if the excision margins are negative [7,18,80]. In patients with multifocal carcinomas, it is recommended to specify in the histopathological report the size of the tumor focus with an indication of the size of the largest focus and the number and sizes of the smaller foci. In the case of CEPA, it is necessary to specify the distance from the carcinoma to the capsule and the specification, to clarify whether it is an intra/extratumoral carcinomatous area. For CEPA, the cut-off point of the extent of the invasion is controversial. The 2005 edition stated that a CEPA with a wider invasion than 1.5 mm from the capsule is an aggressive tumor with poor prognosis. CEPAs less than 4–6 mm from the pleomorphic adenoma border were considered in the past to be minimally invasive carcinoma. Still, for some, cut-off points of 4, 5, 6 and even 8 mm are in discussion [7,8,17].

Another aspect that must be specified in the pathology report is the distance to the nearest excision margin, the histopathological type of carcinoma developed into a pleomorphic adenoma and the size of the invasion. To rule out extracapsular tumor invasion, the entire specimen must be processed and analyzed microscopically [18].

## 5. Future Perspectives

### 5.1. The Tissue-Based Diagnosis

There is a constant evolution of the classification system for the salivary gland neoplastic lesions. Over the years, the WHO classification has refined the categories of benign and malignant salivary gland tumors with the objective of improving the clinical relevance and research feasibility. However, some issues are still pending resolution. Metastasing PAs have to be treated with caution because of their potential aggressive behaviour [17]. The identification of a specific molecular signature for salivary gland tumors is still a growing field. This is due probably to the small number of tumors diagnosed in one center. However, molecular studies have shown that the tumors with oncocytic cells can be variants of salivary neoplastic lesions and not only oncocytomas or oncocytic carcinomas [16]. Hence, oncocytic appearance is seen now as a feature that can be present in various salivary gland lesions, either benign or malignant.

The FNA cytologic diagnosis is invasive and limited by the quality of the sample and the experience of the cytopathologist in head and neck lesions. Furthermore, the FNA technique can be accompanied by complications such as spreading of tumor cells, local recurrence, risk of infection and haemorrhage at the needle puncture site and facial nerve injury [5,54,55,56,57,58,59]. It has been proposed that the MSRSGC malignant category be divided into low-grade and high-grade malignancy and a separate category be used for haematological malignancies [55].

A field which is growing in head and neck neoplasm diagnosis is the liquid biopsy. Several body fluids are analyzed for early detection of tumors. Recent oncology research is focusing on analyzing circulating tumor cells in the peripheral blood. EPISPOT (epithelialimmunospot) assay was proven to be successful in detecting circulating tumor cells in breast cancer, colon cancer, prostate cancer and melanomas. In the subject of liquid biopsy in salivary gland neoplasms, only a few pilot studies on ACC, MEC and SDC cases were published [81,82,83].

MicroRNAs (miRNA) and circulating tumor genes (ctDNA) have been identified in saliva exosomes through quantitative real-time polymerase chain reaction (qRT-PCR), microarray hybridization and sequencing techniques. The expression of miRNA was reported in blood malignancies and solid tumors. In patients with salivary gland neoplasms, miRNA’s role was studied in cases with MEC and ACC. The results of these studies suggested that it may play a role in salivary gland tumor pathogenesis and prognosis [83]. Salivary exosomes could represent the foundation for the development of targeted tumor therapy [84]. Future clinical trials are needed for liquid biopsy to be used as a diagnostic tool in identifying these neoplasms [81,82,83].

### 5.2. Treatment

Commonly, the treatment of malignant salivary gland tumors is surgical, with removal of the tumor without damaging the facial nerve. Adjuvant radiotherapy and chemotherapy show a better regional control but the overall survival rate is not significantly improved. Clinical trials are now being conducted to identify targeted therapies [77,85]. Therapeutic agents targeting transmembrane tyrosine kinase receptors ErbB1 such as Cetuximab have been approved for the treatment of conventional squamous cell carcinoma of the head and neck and are being tested for their efficiency in salivary glands ACC, MEC, MC and AclC [4]. ErbB1-targeted therapy with Gefitimid and the selective inhibition of NF-kB activity with Bortezomid in patients with ACC has showed promising results [4]. Targeting the NOTCH1 mutation in ACC with the inhibitor brontictuzumab produced only a partial response under this therapy [20]. Patients with salivary cancer overexpressing ErbB2 (of another member of the tyrosine kinase receptors) or HER-2 have received additional treatment with Trastuzumab. Trastuzumab is a treatment frequently used for HER-2-positive breast cancer in tumors overexpressing immunohistochemically HER-2 protein or HER-2 gene amplification (detected by fluorescence in situ hybridization). Patients with MEC had partial response to Trastuzumab therapy and patients with SDC showed stabile disease after it. Antiangiogenic agents like Axitinib were tested on ACC but the study was limited by the small number of patients with salivary neoplasia enrolled [4]. Specific target therapeutical agents are still tested on salivary gland tumors but there is no standard treatment for these tumors. Some of the studies showed encouraging data.

Immunotherapy targeting programmed cell death protein 1(PD-1)/programmed cell death 1 ligand 1(PD-L1) and cytotoxic T-lymphocyte-associated protein 4 (CTLA-4) have been used for the treatment of solid tumors. However, few clinical trials have been performed on salivary glands tumors. Furthermore, the number of cases and the tumor types included in those trials were few. Patients with AclC, SDC, MEC, ACC and UC and with progressive diseases were included in trials with PD-1 and PDL-1 inhibitor therapies. The results of those studies showed that for salivary gland tumors, immunotherapy can be promising in some histological types, but in aggressive tumors like ACC no effect was noticed. These studies are limited, and only small samples size were analyzed from just some histological subtypes of salivary neoplasms. At this time, the effectiveness of immunotherapy on salivary glands tumors is considered elusive and the studied patients showed no complete responses to the treatment [86,87].

Non-surgical treatments such as microwave ablation, radiofrequency ablation and ultrasound-guided ethanol sclerotherapy are proposed as options for the management of WTs. However, the experience with these non-surgical treatments is limited. The studies lacked case controls and included a small number of patients with a 6–12 months follow-up. Further studies are needed to appreciate the effectiveness of these options of treatment for WT [88,89].

### 5.3. Artificial Intelligence

**Figure 4:**
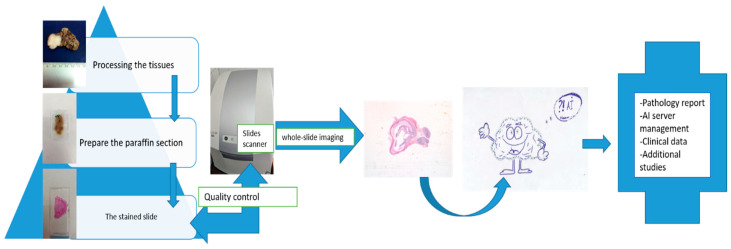
Pathology department AI workflow.

AI algorithms can increase the effectiveness of the pre-surgical diagnosis. However, machine learning diagnostic tools face challenges of algorithm validation, ethics, regulation and difficulty for humans to understand how these artificial neural networks actually make decisions [74,78,90,91,92]. AI analysis algorithms are based on quantitative and qualitative data analysis. For this analysis, numerous data from different diagnosis centers must be included in the study to avoid overfitting. Furthermore, because the data are images, a standardized quality of these images is needed for a higher diagnostic accuracy [71]. In pathology there are some additional steps that are important. For example, the methods of preparing the slide: to have a useful image to analyze the fixation of the tissue, the cutting into sections (e.g., thickness), staining methods and the computer system have to be standardized. The studies that analyze salivary gland tumors usually have a small amount of data to work with because of the rarity of these lesions. Furthermore, the majority of AI-based computed studies are only examining parotid gland tumors; the other major salivary glands and minor salivary glands are not included. If the digital images used to train the computers are manually extracted, then educated pathologists in lesions of head and neck regions have to be involved. Augmentation techniques are used in the AI studies to artificially increase the amount of data by generating new data from the existing data [70]. Because the new data are based on the datasets from the same center, overfitting remains an issue. New datasets are difficult to obtain because of the rarity of the salivary gland tumors. In addition, the need for high-quality images to be analyzed to best characterize the anatomy of the gland or a specific lesion is still there. Histological AI algorithms need to analyze color images, which will generate billions of pixels of data to be processed per patient. For this, specialized AI diagnostic schemes and storage possibilities must be developed [93]. A summary of the steps needed for the application of AI in pathology is shown in Figure 4.

## 6. Conclusions

Diagnosing salivary gland tumors with histopathological examinations, studies represented by MRI, CT or ultrasonography rely on the experience of the medical staff handling the examination tools and clinical data. In the new WHO classification, entities have been combined into broader categories, others were introduced or a specific grade was removed. The high-grade transformation of a salivary gland tumor or the malignant component of the CEPA are features that have to be presented in the pathologist`s examination report. The results of the studies, based on the molecular abnormalities identified in salivary gland neoplasia, will be taken into account when the future classification of these tumors is made. Tumors with unique molecular alteration will be classified according to their genetic features. However, diagnosis challenges can be raised if two different histological types prove to have the same molecular alteration and similar morphology. Currently, molecular studies show that oncocytic carcinomas can be variants of salivary gland carcinoma and not necessarily a specific entity. Furthermore, key molecular alterations are used to enter new entities into the classification of salivary gland tumors, refine the diagnosis and improve the treatment. Molecular-targeted therapy is now tested for treatment of salivary cancer. In the future, AI will be of assistance in the medical field more commonly, so pathology laboratories have to adapt the infrastructure and adjust legislation to fit these new tools of diagnosis. These are novelties that the general pathologist and physician have to be aware of.

## Figures and Tables

**Table 1:** Correlations between tumor morphology and immunohistochemical examination [23,24,25,26,27,28,29,30,31,32,33,34,35,36,37,38,39,40,41,42,43,44,45].

Type of Tumor	Morphological Aspects	Immunohistochemistry
PADK	targetoid pattern and streaming of the tumor cells	−p40, +p63, *Ki-67* index < 10 +SOX10
PA	round, oval, epithelioid, plasmacytoid and spindle tumor cells and myxoid, chondroid, mucoid and chondroid stroma	+GFAP, +SOX10, +PLAG1, +/−p40,
ACC	biphasic tumor with ductal and myoepithelial cells; the tumor cells have angulated nuclei, quantitatively reduced cytoplasm and are arranged in tubular, cribriform or/and solid structures	+p63, p40, +CD117, +MCM-1, +SOX10 and *Ki-67* index > 10
MEC	mucous and squamous cells forming solid nests and cystic spaces	Does not have a typical immunohistochemical profile; +CK7, +CK8, +CK18, +CK19, −SOX10, −CD10
CCC	clear cells	+p63 (diffuse), −CD10
OC	radiologically intraosseous clear cell tumor	−CK7, −CK8, + CK19, −CD10, *Ki-67* index > 5
IC	limited to the salivary gland duct; bordered by myoepithelial cells	the myoepithelial cells +p63, +calponin, +CK14 and +SMA
MC	invasive tumor cells with clear cells, epithelioid, plasmacytoid cytoplasm and splindle cells forming nests, glands or cords	+CK7, +CK14, +vimentin, +S100, +SOX10 +GFAP-15

PADK = polymorphous adenocarcinoma; PA = pleomorphic adenoma; ACC = adenoid cystic carcinoma; MEC = mucoepidermoid carcinoma; CCC = clear cell carcinoma; OC = odontogenic carcinoma; IC = intraductal carcinoma; MC = myoepithelial carcinoma; GFAP = glial fibrillary acidic protein.

**Table 2:** Useful markers in the immunohistochemical diagnosis of benign salivary gland tumors [3,4,5,6,7,8,9,23,24,25,26,27,28,29,30,31,32,33,34,35,36,37,38,39,40,41,42,43,44,45,46].

	Ck	CK7	SMA	Calponin	S100	GFAP	CEA	EMA	p63	Vimentin	CD117	SOX-10
PA	+	US	+	+	+	+	US	US	+	−/+	+	+
BA	+	+	+/−	US	+	−	+	+	+	+	−/+	+
WT	US	US	−	US	−	−	US	US	US	−	US	US
Onc	+	US	+	+	US	+	US	US	+	US	US	−
Myo	US	US	+	+	−/+	−/+	US	US	+	US	US	US
DP	US	+	−	US	−/+	US	+	+	US	−	US	−
SP	+	+	US	US	+	US	+	+	US	+	US	US
IDA	US	+	US	US	+	US	US	US	US	US	US	US
SDA	US	+	−	US	+	US	US	US	−/+	US	US	US
CA	+	US	−	−	+	−	US	US	−	−	+	US

Ck = cytokeratins AE1/AE3; CK = cytokeratin; PA = pleomorphic adenoma; BA = basal cell adenoma; WT = Warthin tumor; Onc = oncocytoma; Myo = myoepithelioma; DP = ductal papilloma; SP = sialadenoma papilliferum; IDA = intercalated duct adenoma; SDA = striated duct adenoma; CA = canalicular adenoma; US = unspecified; − = negative; + = positive.

**Table 3:** Common immunohistochemical examination in malignant tumors of the salivary glands [3,4,5,6,7,8,9,23,24,25,26,27,28,29,30,31,32,33,34,35,36,37,38,39,40,41,42,43,44,45,46,47,48,49].

	Ck	CK7+/ CK20−	CK8	CK19	EMA	SMA	Vim	S100	GFAP	AR	PR	ER	p63	PSA	CD117	*Ki-67*	CEA
MEC	+	YES	+	+	+	−/+	−/+	−/+	−/+	−	US	−	+	US	US	US	−/+
AclC	+	YES (with some CK7-/CK20+)	+	US	+	−	US	−/+ (foc)	US	US	US	US	−	US	US	>5%	+
ACC	+(L)	YES	US	+	+(L)	+(NL)	+	+	−/+	−/+	−/+	−/+	+(în NL)	US	+	>10% 13.6–34.7%)	+(L)
PADK	+	US	US	US	+	+/−	+	+	−/+	US	US	US	+	US	US	<10%	+
BCADK	+	US	+	+	+ (foc)	−/+ (foc)	+	+ (foc)	US	+	US	US	US	US	+	US	+ (foc)
SDC	+	YES	US	US	+	−	−/+	−/rarely +	+	+	−	−	−	−/+	US	>10% (2.7–50% of cases)	+
CCC	+	−	−	+	+	−	−	−/+	−	US	US	US	+	US	US	US	+
EMC	+(L)	US	US	US	+	+	+	+ (myo cells)	US	+(L)	US	US	+	US	+	−/+	−
MC	+	+	US	US	−/+	+/−	+	+	+	US	US	US	−/+	US	−/+	>10%	−

Ck = cytokeratins AE1/AE3; MEC = mucoepidermoid carcinoma; AclC = acinic cell carcinoma; PADK = polymorphous adeocarcinoma; BADK = basal cell adenocarcinoma; SDC = salivary duct carcinoma; CCC = clear cell carcinoma; EMC = epithelial-myoepithelial carcinoma; MC = myoepithelial carcinoma; GFAP = glial fibrillary acidic protein; EMA = epithelial membrane antigen; CEA = carcinoembryonic antigen; AR = androgen receptors, PR = progesterone receptors; US = unspecified; L = luminal cells; NL = nonluminal cells; − = negative; + = positive, and foc = focally.

## Data Availability

Data are contained within this review. No new data were created or analyzed in this study.
